# Mucinous tubular and spindle renal cell carcinoma revealed by a trauma of the kidney: a case report

**DOI:** 10.1186/s13256-024-04659-0

**Published:** 2024-07-12

**Authors:** Kays Chaker, Mahdi Marrak, Nader Gharbia, Alia Zehani, Yassine Ouanes, Yassine Nouira

**Affiliations:** 1 Department of Urology, LA RABTA Hospital, University of Tunis El Manar, Tunis, Tunisia; 2 Department of Pathology, LA RABTA Hospital, University of Tunis El Manar, Tunis, Tunisia

**Keywords:** Mucinous, Renal cell carcinoma, Spindle, Tubular

## Abstract

**Background:**

Mucinous tubular and spindle cell carcinoma is a rare renal tumor. It has been recognized as a distinct entity in the 2004 World Health Organization tumor classification. Since then, several dozen of these tumor have been reported with additional complementary morphologic characteristics, immunohistochemical profile, and molecular genetic features that have further clarified its clinicopathologic aspects.

**Case presentation:**

We report the case of a 52-year-old male African patient who was found to have a mucinous tubular and spindle renal cell carcinoma on a nephrectomy specimen for a severe kidney trauma.

**Conclusions:**

This tumor has a histological spectrum ranging from low to high grade, which includes sarcomatoid differentiation that can confer the tumor an aggressive clinical course.

## Background

Mucinous tubular and spindle cell renal cell carcinoma is a rare and recently described variant of renal cell carcinoma [1]. It has been classified as a separate entity in the 2004 World Health Organization tumor classification [1]. Because mucinous tubular and spindle cell renal cell carcinoma is rare, references are limited. To the best of our knowledge, there are no unified diagnostic criteria, especially for the imaging diagnosis of mucinous tubular and spindle cell renal cell carcinoma or the outcome of this variant of renal cancer. Therefore, it is necessary to collect more clinical and imaging characteristics to improve the diagnosis and treatment.

## Case presentation

**Fig. 1:**
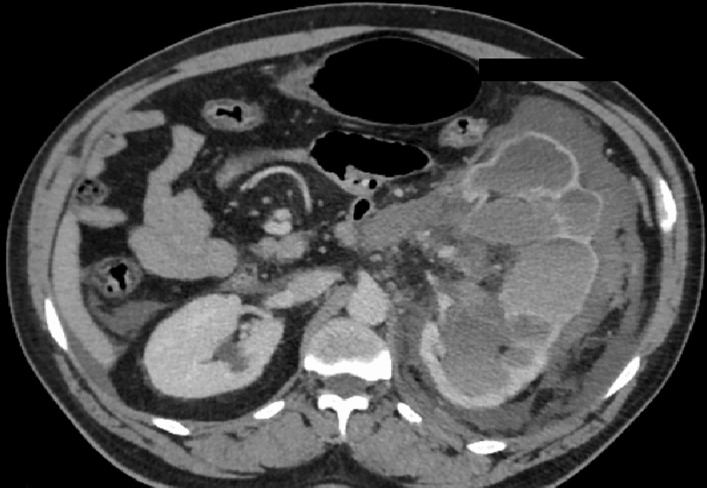
Abdominal computed tomography scan showed a high-volume retroperitoneal hematoma and multiply lacerated lower pole of the left kidney

**Fig. 2:**
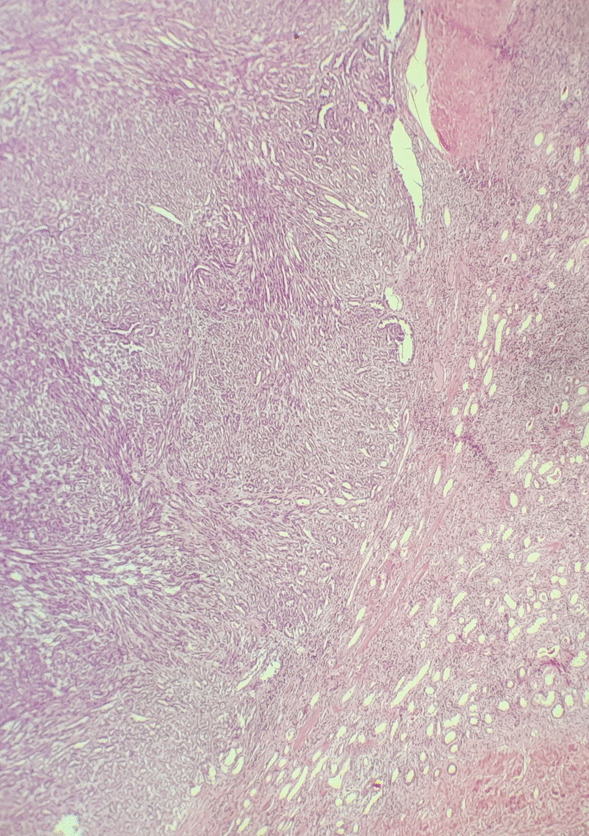
Renal parenchyma with tumor proliferation comprising tubular structures and a fusocellular contingent (hematoxylin–eosin ×10)

**Fig. 3:**
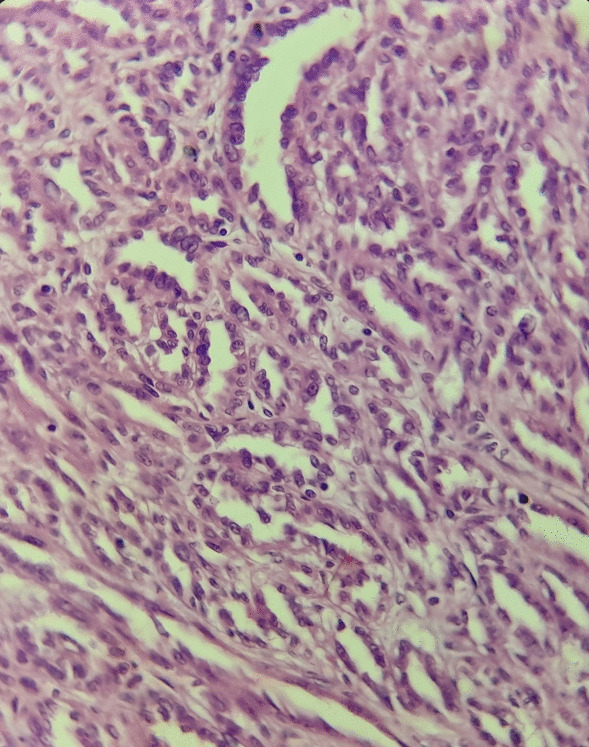
Elongated tubular structures bordered by cubic cells with low-grade nuclei (hematoxylin–eosin ×40)

A 52-year-old male African patient with no prior medical or surgical history consulted emergencies for a left flank pain and hematuria after falling from his own height and onto his left flank. The patient had a blood pressure of 100/50 mmHg, a heart rate of 135 beats per minute, a respiratory rate of 20, and oxygen saturation of 94%. Physical examination revealed tenderness at the left flank region. Contusions and ecchymoses were absent. Initial laboratory evaluation revealed hemoglobin of 8.7 g/dl. Cytobacteriological examination of the urine showed the presence of macroscopic hematuria with a negative culture. Other tests including coagulation, ionogram, and creatinine were within normal limits. Abdominal computed tomography scan showed a high-volume retroperitoneal hematoma and a multiply lacerated lower pole of the left kidney (Fig. 1). Preoperatively, 4 units of blood were transfused. Urgent open surgery was performed. Intraoperatively, there was a large retroperitoneal hemorrhage, and the dissection of the kidney was difficult. A left radical nephrectomy was done by lombotomy. After surgery, the patient was transferred to the intensive care unit for a 3-day follow-up, and clinical improvement began on the third postoperative day. The patient was discharged without incident on the seventh postoperative day after removal of the redon and with a normal biological check-up. Histological findings were consistent with mucinous tubular and spindle cell renal cell carcinoma of the left kidney (Figs. 2 and 3). After 12 months of clinical and radiological (abdominal, pelvic, and thoracic computed tomography scans) follow-ups, there was no functional complaint or any sign of recurrence.

## Discussion

Several isolated cases or small series of mucinous tubular and spindle cell renal cell carcinoma have been reported [2]. Although some forms are symptomatic [1], the majority are discovered incidentally during abdominal imaging examinations performed for other clinical reasons. Radiologically, mucinous tubular carcinoma and spindle cell renal cell carcinoma share a common appearance reminiscent of the scanographic appearance of papillary renal cell carcinoma [1, 3]. Histologically, the tumor is characterized by the presence of tubular and spindle-shaped cells separated by a mucinous stroma [4]. Nuclei are usually round and uniform with low nuclear density, but a few forms of high nuclear grade may occasionally be observed. [1]. Examples of mucinous tubular and spindle cell renal cell carcinoma with sarcomatoid differentiation have been recently reported [1]. The oncological prognosis of this renal tumor is generally favorable, given its low-grade malignancy, and complete surgical excision appears to be the appropriate treatment [5]. The rare metastases reported are generally due to high-grade malignancies or sarcomatoid forms [5]. The majority of patients reported in the previous studies [6] were metastasis free, with only a few patients [3] presenting with pulmonary metastases, bone metastases, and lymph node metastases, which indicates that mucinous tubular and spindle cell renal cell carcinoma is an indolent renal cancer with a low mortality rate.

## Conclusions

Mucinous tubular and spindle cell renal cell carcinoma is rare and only relatively recently described, and thus no international consensus exists with regard to patient outcomes or optimal follow-up for this diagnosis. Further molecular studies are needed to clarify the histogenesis of this tumor.

## Data Availability

The datasets are available from the corresponding author on reasonable request.
